# The oncometabolite R-2-hydroxyglutarate inhibits microglial activation via the FTO/NF-κB pathway

**DOI:** 10.3389/fonc.2025.1525761

**Published:** 2025-11-24

**Authors:** Lu Wang, Huiting Zhang, Xifeng Jing, Yuanchi Huang, Yuxin Kang, Yulan Qian, Dong Chen

**Affiliations:** 1 Hematology Center, Cyrus Tang Medical Institute, Jiangsu Institute of Hematology, Collaborative Innovation Center of Hematology, National Clinical Research Center for Hematological Diseases, Soochow University, Suzhou, China; 2 Department of Pharmacy, The First Affiliated Hospital of Soochow University, Suzhou, China; 3 Ministry of Education (MOE) Engineering Center of Hematological Disease, Soochow University, Suzhou, China

**Keywords:** microglia, R-2HG, glioma, NF-κB, FTO

## Abstract

**Introduction:**

Mutations in isocitrate dehydrogenase 1 (mIDH1) generate the oncometabolite (R)-2-hydroxyglutarate (R-2HG), which promotes tumorigenesis by inhibiting α-ketoglutarate-dependent enzymes and altering the epigenetic landscape. Microglia, the resident brain macrophages, are a key immune population in gliomas. While R-2HG is known to impair CD8+ T-cell function, its specific impact on microglial activation remains unknown. This study aimed to investigate the effect of R-2HG on microglial inflammatory responses.

**Methods:**

The murine microglial BV2 cell line was stimulated with glioma-conditioned medium (CM) in the presence or absence of R-2HG. Cytokine production was analyzed, with a specific focus on IL-6. Mechanistic studies involved assessing the phosphorylation and nuclear translocation of key NF-κB pathway components (IκBα and p65). The dependency on α-ketoglutarate was tested via metabolite supplementation, and the role of the demethylase FTO was investigated.

**Results:**

Treatment with glioma CM significantly induced cytokine production in BV2 cells. R-2HG specifically inhibited the activation of IL-6. Mechanistically, R-2HG suppressed CM-induced phosphorylation of IκBα and p65, thereby impairing the nuclear translocation of p65. The inhibitory effect of R-2HG on IL-6 was abolished by the addition of α-ketoglutarate. Further analysis demonstrated that R-2HG downregulates IL-6 expression by inhibiting the activity of the RNA demethylase FTO.

**Discussion:**

Our findings reveal that R-2HG specifically inhibits microglial inflammatory activation by suppressing the FTO/NF-κB signaling pathway, leading to decreased IL-6 production. This study provides a novel mechanism by which R-2HG modulates the tumor immune microenvironment, which may be beneficial for exploring the basis of antitumor immunity in IDH-mutant gliomas.

## 1 Introduction

Gliomas are primary central nervous system (CNS) tumors that are thought to originate from neural stem or progenitor cells carrying genetic alterations (1).With advances in molecular biology and sequencing technologies, significant development has been made in the key molecular alterations of gliomas (2). The 2021 WHO classification of central nervous system tumors classifies adult diffuse gliomas into three different types: astrocytoma, IDH mutant; oligodendroglioma, IDH mutant and 1p/19q co-deleted; glioblastoma, IDH wild-type. These mutations are regarded as an early event in the progression of low-grade gliomas to higher-grade gliomas (2, 3).

The R132H mutation of IDH1 is the most common in gliomas, accounting for approximately 90% of cases (4). Mutations at this position alter the activity of the IDH1 enzyme, which converts α-ketoglutarate (α-KG) to R-2-hydroxyglutarate (R-2HG), leading to the accumulation of R-2HG at millimolar levels in tumors (5, 6). R-2HG has been identified as an oncometabolite, driving malignant transformation and tumor progression. Additionally, as an α-KG analog, R-2HG competitively inhibits α-KG-dependent enzymes (7), including JmjC family histone demethylases, TET family DNA demethylases, and RNA demethylases such as fat mass- and obesity-associated gene (FTO) and ALKBH, which are associated with the altered epigenetic status in IDH-mutant tumors (7–10).

In the glioma microenvironment, the predominant cell type is microglia/macrophages, which comprise approximately 30% of the total tumor mass (11). In microglia, originated from yolk sac-derived progenitor cells, the resident macrophages in the central nervous system are the first responders to brain injury, which produce a range of inflammatory cytokines, including tumor necrosis factor alpha (TNFα), interleukin-6 (IL-6), interleukin-1β (IL-1β), nitric oxide synthase (iNOS), and matrix metalloproteinase (MMP) (12, 13). Microglia are also associated with glioma development, and resident microglia are recruited into tumor tissue at the earliest stages of glioblastoma (GBM), whereas only a small number of macrophages are specifically recruited in the later stages (14). Thus, microglia act as the predominant immune cell population in gliomas at the early stage and may represent as a more efficacious therapeutic target (15).

Recent studies show that R-2HG participates in the process of immunosuppression through a direct paracrine effect on a vast array of immune cells, remodeling the tumor microenvironment (TME) and promoting glioma development (16–18). In comparison with wild-type (WT) gliomas, human and mouse IDH1-mutant gliomas show lower inflammatory cytokine and chemokine expression, and tumor-infiltrating lymphocytes, which suggests that R-2HG facilitates the formation of the immunosuppressive TME (16, 19, 20). However, the precise mechanism by which the IDH1-mutant metabolite, R-2HG, suppresses inflammatory activation in gliomas remains to be elucidated.

In this study, we investigated the role of R-2HG on the activation of microglia in the glioma microenvironment. We found the conditioned medium (CM) from GL261 or ALTS1C1 mouse glioma cell-activated BV2 cells, whereas R-2HG treatment specifically inhibited the production of IL-6. The transcription of IL-6 is mainly dependent on the NF-κB pathway. R-2HG significantly inhibited the nuclear translocation of p65, and downregulating CM induced phosphorylation of IκBα and p65 by inhibiting FTO activity in BV2 cells. Meanwhile, the addition of α-KG could abolish the inhibitory effect of R-2HG on IL-6 but could not in the presence of the FTO inhibitor. Given these findings, novel FTO-targeting therapeutics may be a beneficial addition for IDH mutant gliomas.

## 2 Materials and methods

### 2.1 Reagents and antibodies

Cell-permeable 1-octyl-R-2HG (TFMB-R-2HG) and bacterial lipopolysaccharide (LPS) were purchased from Sigma (St. Louis, MO, USA). The CCK-8 kit was purchased from APExBIO (Houston, TX, USA). The nitric oxide assay kit was obtained from Beyotime (Shanghai, China). D-α-Hydroxyglutaric acid disodium salt (R026713) was purchased from Rhawn (Shanghai, China). Antibodies against iNOS (13120S), p65 (8242S), phosphorylated (p)-p65 (Ser536,3033S), IκBα (4812S), phosphorylated (p)-IκBα (2859S), and histone H3 (4499S) were obtained from Cell Signaling Technology (Beverly, CA, USA). The anti-β-actin antibody (FD0060) was purchased from Hangzhou Fude Biotechnology (Hangzhou, China). FITC-conjugated Affinipure Goat Anti-Rabbit IgG(H+L) secondary antibody (SA00003-2) was purchased from Proteintech (Wuhan, China). Mouse IL-6 Uncoated ELISA Kit (88-7064-77) and Mouse TNFα Uncoated ELISA Kit (88-7324-76) were purchased from Invitrogen (Carlsbad, CA, USA). ELISA stop buffer was purchased from Solarbio (Beijing, China). The reagents and consumables required for cell culture were purchased from NEST (Wuxi, China). BAY 11-7082 (HY-13453), JSH-23 (HY-13982), 666-15 (HY-101120), SR11302 (HY-15870), GSK-J4 (HY-15648B), PFI-90 (HY-139348), KDM2/7-IN-1 (TC-E 5002, HY-107573), and Bobcat-339(HY-111558) were purchased from MedChemExpress (Monmouth Junction, NJ,USA), and selumetinib (S1008) and FB23-2 (S8837) were purchased from (Houston, TX, USA), respectively.

### 2.2 Cell culture

The BV2 microglial cell line was a gift from Dr. Wang Guanghui (Soochow University, Suzhou, China). GL261 cells were obtained from Procell (Wuhan, China). The ALTS1C1 cell line was a gift from Dr. Wang Mei (Children’s Hospital, Soochow University, Suzhou, China). BV2 cells, GL261 cells, and ALTS1C1 cells were cultured in Dulbecco’s modified Eagle medium (DMEM; Gibco, Grand Island, NY, USA) containing 10% fetal bovine serum (FBS, Gibco). All cells were incubated in a humidified atmosphere containing 5% CO_2_ at 37°C.

### 2.3 Isolation of mouse primary microglia

Mouse primary microglial cultures were prepared from neonatal C57BL/6 mouse brain tissues according to a previously described protocol with minor modifications (21). Briefly, cerebral cortices from newborn pups were minced, digested with papain and DNase I (both from Sigma-Aldrich), and filtered through a 40-μm cell strainer. The dissociated cells were collected by centrifugation and seeded into poly-D-lysine-coated flasks containing F12 medium (Gibco, New York, USA), followed by incubation at 37°C for 14 days. To isolate microglia, mixed glial cultures were subjected to orbital shaking at 180 rpm for 6 h at 37°C. The detached microglial cells were collected and cultured in appropriate media for subsequent ELISA assays.C57BL/6J mice were supplied by the Center of Experimental Animals of Soochow University and housed in a specific pathogen-free room under controlled temperature and humidity. All mouse procedures were conducted according to the Institutional Guide for the Care and Use of Medical Laboratory Animals and approved by the Ethics Committee of Soochow University (202206A0189).

### 2.4 CCK-8 assay

Cell viability was conducted utilizing the CCK-8 kit (APExBIO, USA), and experiments were finished with the method provided by the manufacturer. A 10-μL CCK-8 solution was added to 96-well plates at the time specified in the manufacturer’s instructions, which were incubated for 2×10^3^ cells, and then the absorbance was measured at 450 nm after continued incubation for 2h at 37°C.

### 2.5 Measurement of the nitric oxide release

The amount of nitric oxide (NO) released was measured using the Griess reagent (Beyotime, China), and experiments were finished following the manufacturer’s protocol. Briefly, 100 μL of cell culture supernatant was mixed with an equal volume of Griess reagent (final concentration of 0.1% N-[1-naphthyl] ethylenediamine dihydrochloride in distilled water and 1% sulfanilamide in 5% phosphoric acid) on a 96-well flat-bottom plate. The absorbance was measured at 540 nm after 10 min. The amount of NO produced was calculated from a standard curve plotted using sodium nitrite.

### 2.6 RNA isolation and quantitative-PCR assays

Total RNA was extracted with the TRIzol reagent, and then 800 ng RNA was reversely transcribed to cDNA. For each qPCR reaction, 40 ng RNA was subjected to qPCR with the SYBR Green system (Vazyme, Nanjing, China) using the specific primers shown in **Supplementary Table S1**. The reaction condition was as follows: 95°C for 5 min, followed by 40 cycles consisting 95°C for 10 s, and 60°C for 30 s. Data were normalized to β-actin expression.

### 2.7 Enzyme-linked immunosorbent assay

Culture supernatants of BV2 cells were collected after treatments. Levels of secreted IL-6 and TNFα were detected according to the ELISA Kit manufacturer’s instructions (Invitrogen, San Diego, CA, USA). The capture antibody (100 μL, 1:250) was added to a 96-well microplate and incubated with the coating buffer (carbonate buffer, 50 mM, pH 9.6) at 4°C overnight. Then, the coating buffer was removed, and the microplate was washed three times with the wash buffer (PBS containing 0.05% Tween 20, 10 mM, pH 7.4). The microplate was then blocked with 5% skimmed milk (300 μL) at room temperature for 2 h. Next, the standard and samples were added to the antibody-coated 96-well plates (100 μL/well) and incubated at room temperature for 2 h. Then, the plates were washed with PBST five times and patted on a filter paper. After the addition of 100 μL of the biotinylated complex to each well and an incubation for 1 h, the plate was washed with PBST five times. A horseradish peroxidase-labeled avidin complex (100 μL) was added and incubated at room temperature for 30 min before five washes with PBST. Enzyme reaction substrate (100 μL) was added and incubated for 10 min. The reaction was terminated by the addition of ELISA stop buffer, and then the OD value was measured at 450 nm using a microplate reader.

### 2.8 Preparation of nuclear and cytosolic fractions

Nuclear and cytosolic fractions were prepared using the Nuclear and Cytoplasmic Protein Extraction Kit according to the manufacturer’s instructions (Beyotime, Shanghai, China). Cells were homogenized and lysed with 200 μL ice-cold Cell Plasma Protein Extraction Reagent A, and then bathed with ice for 15 min; next, 10 μL Cytoplasmic Protein Extraction Reagent B was added, vortexed for 5 s, and bathed with ice for 1 min, followed by centrifuging for 5 min at 10,000G. The cytosolic extracts were collected and stored at −80 °C. The nuclear pellet was resuspended and incubated with 50 μL Nuclear Protein Extraction Reagent for 30 min. The nuclear lysate was centrifuged for 10 min at 10,000G, and the fraction containing the soluble nuclear proteins was stored at −80 °C until further assay.

### 2.9 Western blotting

Cells were harvested in RIPA lysis buffer [150 mM NaCl, 50 mM Tris–HCl, pH 8.0, 1% Triton X-100, 0.5% sodium deoxycholate, 0.1% sodium dodecyl sulfate, and protease inhibitor cocktail tablets (MedChemExpress, USA)]. After determining the protein concentration, 20 μg of each sample was loaded onto sodium dodecyl sulfate-polyacrylamide gel electrophoresis gels, electrophoresed, and transferred to 0.2-μm polyvinylidene difluoride membranes (Millipore, Billerica, MA, USA). Membranes were blocked with 5% non-fat milk in Tris-buffered saline (TBS) for 2 h and then exposed to specific primary antibodies and the β-actin antibody overnight at 4°C. The membranes were then incubated with secondary antibodies (Immunoway) after three washes with TBS containing 0.1% Tween-20 (Sigma-Aldrich) (TBST). A Tanon Chemi Dog 5200T (Tanon, Shanghai, China) instrument was used to visualize the protein bands according to the manufacturer’s instructions.

### 2.10 Immunofluorescence staining

Cells were grown on a glass-bottom cell culture dish (Nest, Wuxi, China) for 24 h, followed by either conditioned culture stimulation or treatment with conditioned culture and R-2HG. The cells were fixed with 4% paraformaldehyde for 10 min and permeabilized for 10 min with 0.2% Triton X-100. After blocking for 60 min with 1% BSA, cells were incubated with p65 antibody at 4°C overnight. Then, after incubating with FITC-conjugated Affinipure Goat Anti-Rabbit IgG(H+L) secondary antibody for 60 min at room temperature, DAPI (Beyotime, Shanghai, China) was used for nuclear staining. Images were acquired using a confocal laser scanning microscope (Olympus, Japan). Cytoplasmic and nuclear fluorescence intensities were calculated using ImageJ software (National Institutes of Health, Bethesda, MD, USA) and are expressed as a percentage of nuclear and cytoplasmic p65 protein.

### 2.11 Statistical analysis

All statistical analyses were performed using GraphPad Prism 8. Data are presented as the means ± SD. Student’s t-test was used for unpaired comparisons of two groups. Differences between more than two groups were determined by one-way analysis of variance (ANOVA) or two-way ANOVA. p < 0.05 was considered significant.

## 3 Results

### 3.1 R-2HG impairs CM-induced BV2 activation

We initially used TFMB-R-2HG (permeable R-2HG) to treat LPS-stimulated BV2 cells. It was shown that TFMB-R-2HG had no effect on the cell viability of BV2 cells (**Supplementary Figure S1A**) but significantly suppressed the release of NO and the expression of nitric oxide synthase (iNOS) induced by LPS (**Supplementary Figures S1B-D**). Meanwhile, we examined other pro-inflammatory cytokines and found that TFMB-R-2HG significantly inhibited the synthesis and secretion of IL-6, TNFα, and IL-1β in activated BV-2 microglial cells (**Supplementary Figures S1E-I**). These results indicated that TFMB-R-2HG inhibited the inflammatory activation markers that we measured in BV2 cells stimulated by LPS.

While the cell-permeable TFMB-R-2HG was a crucial tool for establishing the intrinsic capacity of R-2HG to suppress microglial activation, its utility for modeling the glioma microenvironment is limited. The maximum solubility of TFMB-R-2HG (867 mM in DMSO) prevents its application at the high millimolar concentrations that are physiologically relevant for IDH-mutant gliomas (up to 30 mM) (5, 22). To more accurately recapitulate these conditions, we employed unmodified R-2HG disodium salt. Although impermeable, this natural form of the oncometabolite is highly soluble, allowing us to treat cells at physiological doses. Critically, its activity is dependent upon cellular uptake via specific transporters. We confirmed that glial cells, including microglia, highly express the dicarboxylic acid transporter SLC13A3 (**Supplementary Figure S2**), which has previously been shown to mediate the uptake of R-2HG into human T cells at a concentration of up to 4–15 mM (17). Therefore, the use of R-2HG disodium salt provides a more physiologically relevant model to study the paracrine effects of glioma-derived R-2HG on microglia in the glioma microenvironment.

**Figure 1:**
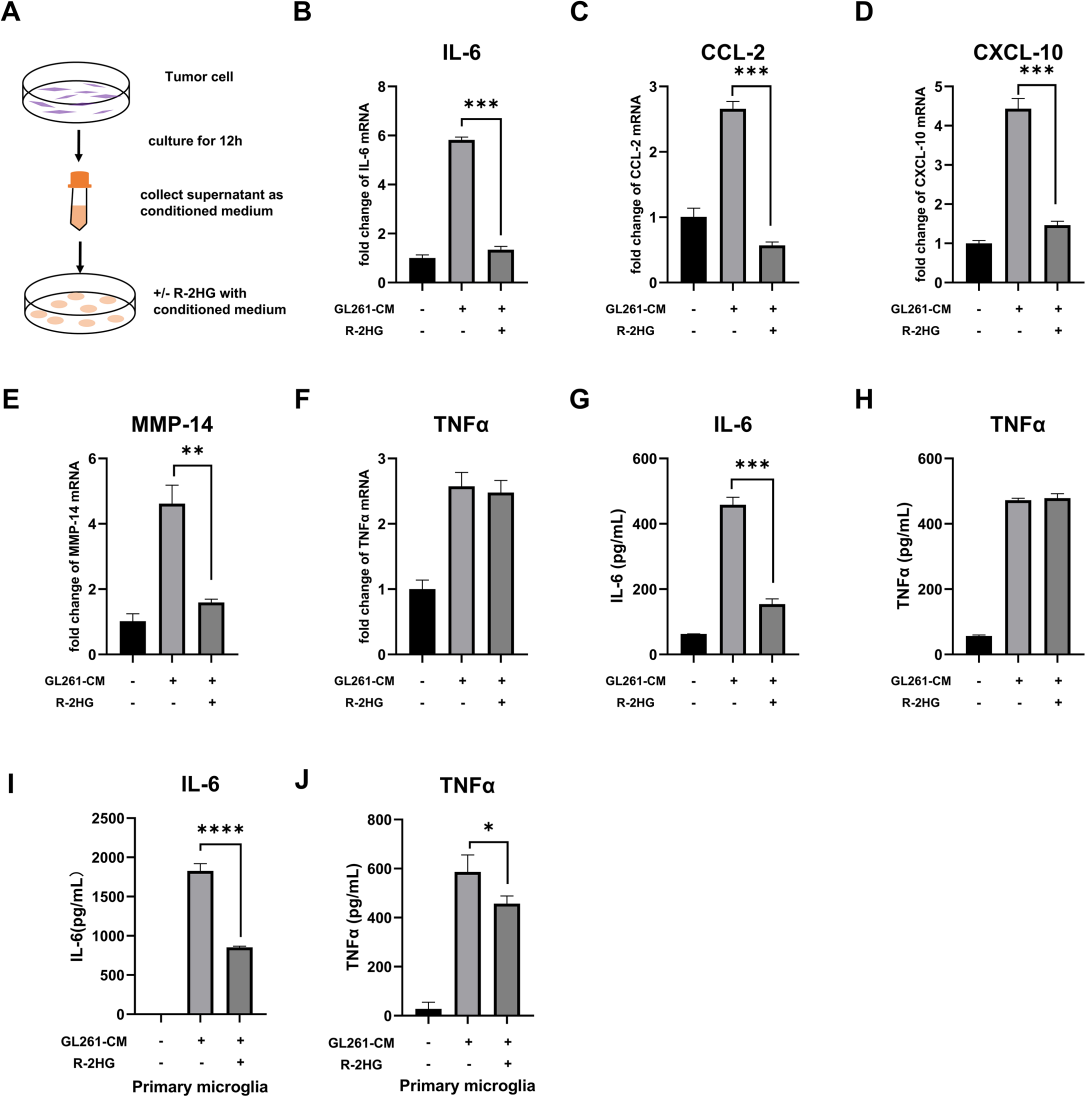
R-2HG dampens GL261-CM-induced inflammatory activation in the BV2 cell. **(A)** Schematic of the glioma-conditioned medium system. 2.5×10^6^ GL261 or ALTS1C1 glioma cells were plated in a 10-cm dish with 10 ml medium; when the cell density reaches 80%, the medium was replaced with another 10-ml fresh medium and the glioma cells were cultured for 12 h, and then the supernatant was collected as the conditioned medium for subsequent experiments. **(B-F)** BV2 cells were treated with conditioned medium from GL261 glioma cells and 20 mM R-2HG; after incubation for 6 h, the mRNA expression levels of IL-6, CCL-2, CXCL-10, MMP-14, and TNFα were determined using quantitative real-time PCR (qPCR). **(G, H)** BV2 cells were incubated with conditioned medium from GL261 glioma cells and 20 mM R-2HG for 24 h, and the supernatants were collected to determine the protein expression levels of IL-6 and TNFα, measured by enzyme-linked immunosorbent assays (ELISAs). **(I, J)** Primary microglia were incubated with conditioned medium from GL261 glioma cells and 20mM R-2HG for 24h, and the supernatants were collected to determine the protein expression levels of IL-6 and TNFα, measured by ELISAs. Data are presented as the means ± SD (n = 3) and are representative of three independent experiments. Student’s t-test was used for unpaired comparisons of two groups. *p < 0.05, **p < 0.01, ***p < 0.001, and ****p < 0.0001 compared with CM.

To investigate the specific effect of R-2HG on glioma-associated inflammation, we established a model of microglial activation. We collected conditioned medium (CM) from IDH-wild-type GL261 and ALTS1C1 mouse glioma cells (which do not produce R-2HG) to mimic the soluble pro-inflammatory signals found in a generic tumor microenvironment. As expected, the conditioned medium from either GL261 or ALTS1C1 glioma cells significantly activated BV2 cells with a notable increase in pro-inflammatory cytokines, including IL-6, CCL-2, CXCL-10, MMP-14, TNFα, IL-1β, and iNOS (**Figures 1A–H**, **Supplementary Figures S3A-C**, **S4A-J**). We then supplemented this CM with R-2HG to mimic the TME of IDH-mutant glioma. The data showed that R-2HG treatment specifically reduced the mRNA levels of IL-6, CCL-2, CXCL-10, and MMP-14 in CM-activated BV2 cells (**Figures 1B-E**, **Supplementary Figures S4A-D**), whereas the mRNA levels of TNFα, IL-1β, and iNOS were not reduced (**Figure 1F**, **Supplementary Figures S3A-B**, **S4E-G**). The protein levels of these genes checked by ELISA show similar results; R-2HG selectively suppressed the production of IL-6, without influencing TNFα and iNOS in BV2 cells stimulated by CM (**Figures 1G-H**, **Supplementary Figures S3C**, **S4H-J**). Furthermore, we isolated primary microglia from mice and observed that conditioned medium derived from GL261 cells significantly stimulated the secretion of cytokines IL-6 and TNFα, whereas R-2HG markedly suppressed their production in primary microglia (**Figures 1I, J**). Results indicate that R-2HG can impair pro-inflammatory responses in microglia, providing a mechanistic basis for the less inflammatory tumor microenvironment observed in IDH-mutant gliomas. Specifically, R-2HG significantly downregulates the expression of key pro-inflammatory cytokines, including IL-6.

### 3.2 R-2HG inhibits CM-induced NF-κB signaling pathway activation in BV2 cells

**Figure 2:**
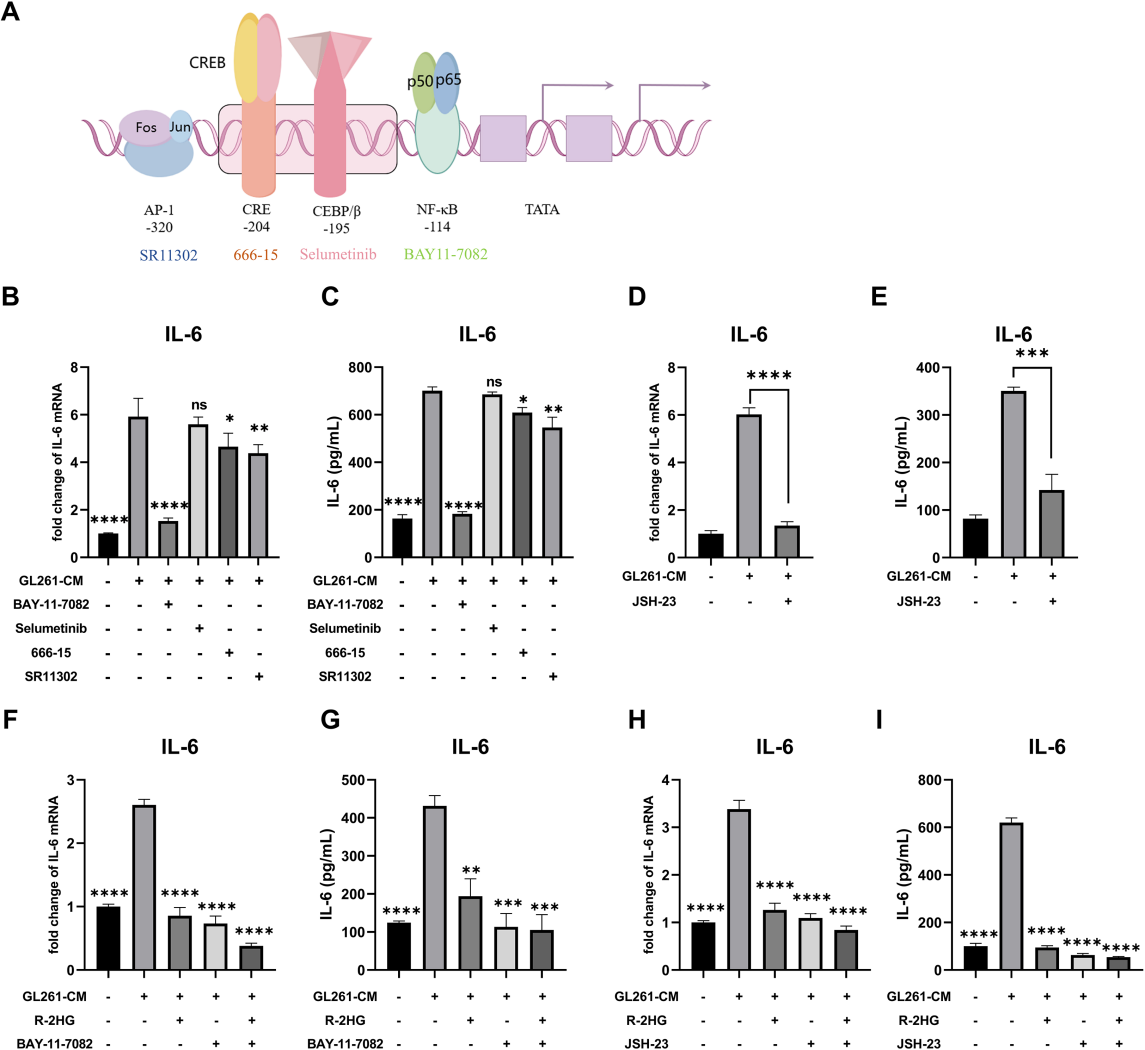
R-2HG attenuates IL-6 production by inhibiting the NF-κB pathway in GL261-CM-induced BV2 cells. **(A)** Schematic representation of IL-6 transcription factors, such as NF-κB, CEBP/β, CREB, and AP-1. **(B, C)** Stimulated with conditioned medium from GL261 glioma cells, BV2 cells were treated with inhibitors of different transcription factors of IL-6; the cell pellets and cell supernatants were analyzed for mRNA and protein expression levels of IL-6 using qPCR and ELISA, respectively. In addition, BAY11-7082 (5 μM) is an inhibitor of NF-κB, selumetinib (5 μM) is an inhibitor of MEK (mitogen-activated extracellular signal-regulated kinase), the upstream protein of CEBP/β, since there is no inhibitor that acts directly on CEBP/β, the inhibitor of MEK was used as an alternative pathway, 666-15 (1 μM) is an inhibitor of CREB, and SR11302 is an inhibitor of AP-1. **(D, E)** BV2 cells were treated with another NF-κB inhibitor, JSH-23 (10 μM); under the stimulation of conditioned medium from GL261 glioma cells, the cell pellets and cell supernatants were collected at 6 or 24 h and assayed for IL-6 mRNA and protein expression levels using qPCR and ELISA, respectively. **(F, G)** Stimulated by conditioned medium from GL261 glioma cells, BV2 cells were treated with R-2HG (20 mM) or BAY11-7082 (5 μM) or cotreated with R-2HG (20 mM) and BAY11-7082 (5 μM) for 6 or 24 h, and then the mRNA and protein expression levels of IL-6 were examined respectively. **(H, I)** BV2 cells were treated with R-2HG (20 mM) or JSH-23 (10 μM) or cotreated with R-2HG (20 mM) and JSH-23 (10 μM) for 6 or 24 h, and then the mRNA and protein expression levels of IL-6 were examined, respectively. Data are presented as the means ± SD (n = 3) and are representative of three independent experiments. Student’s t-test was used for unpaired comparisons of two groups. Differences between more than two groups were determined by one-way ANOVA with a Dunnett’s *post-hoc* test. *p < 0.05, **p < 0.01, ***p < 0.001, and ****p < 0.0001 compared with CM. "ns" indicates a non-significant result (p ≥ 0.05).

Since R-2HG inhibited CM-activated IL-6 transcription, we sought to identify which transcription factor pathway was responsible for its activation and was therefore being targeted by R-2HG. Previous studies have found some major transcription factors of IL-6 (**Figure 2A**): the nuclear factor kappa-B (NF-κB) (23), CCAAT-enhancer-binding proteins β (CEBP/β) (24), cAMP response element binding protein (CREB) (25), and activator protein-1 (AP-1) (26). Thus, the inhibitors of these transcription factors were used to treat CM-activated BV2 cells. The results showed that NF-κB inhibitor BAY11–7082 significantly decreased the mRNA and protein expression levels of IL-6 in BV2 cells activated by CM from GL261 or ALTS1C1 glioma cells (**Figures 2B, C**, **Supplementary Figure S4A-B**). Meanwhile, another NF-κB inhibitor, JSH-23, was employed to confirm our finding. The JSH-23 treatment also notably reduced IL-6 expression in CM-activated BV2 cells (**Figures 2D, E**, **Supplementary Figures S5C-D**). Based on our results, we confirmed that the NF-κB signaling pathway was primarily responsible for IL-6 transcription in CM-activated BV2 cells. Then, we checked whether R-2HG inhibits IL-6 transcription via the NF-κB signaling pathway. The results showed that the IL-6 activation was significantly inhibited when the BV2 cells were treated with the NF-κB inhibitor (BAY11–7082 or JSH-23) or R-2HG alone. However, the combination of two drugs failed to show further inhibitory effect on IL-6 activation in BV2 cells (**Figures 2F, I**, **Supplementary Figures S5E-H**). Consequently, these findings indicated that R-2HG inhibited IL-6 transcription in activated BV2 cells via the NF-κB pathway.

**Figure 3:**
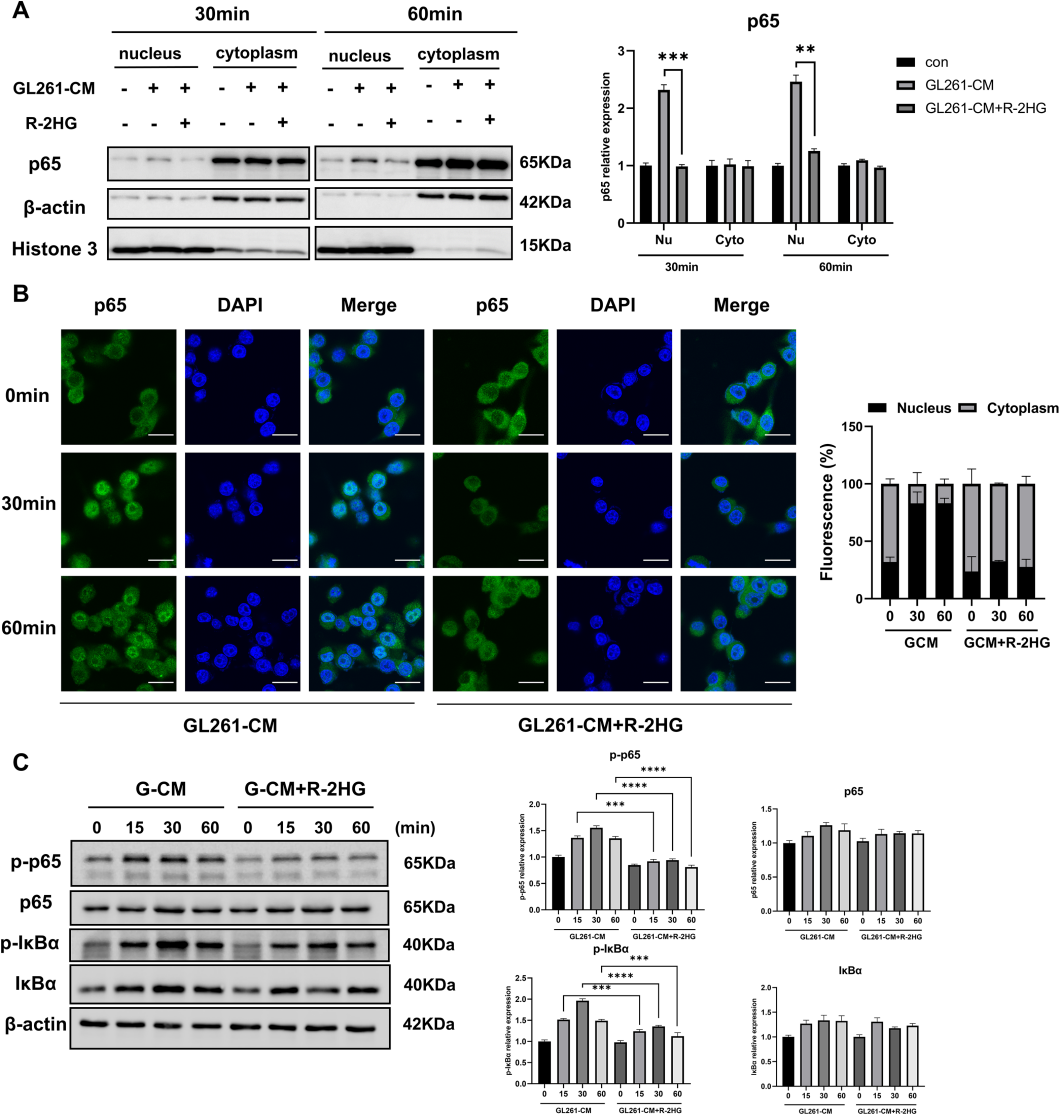
R-2HG reduces p65 phosphorylation and nuclear entry in GL261-CM-induced BV2 cells. Stimulated with conditioned medium from GL261 glioma cells, **(A)** BV2 cells were treated with R-2HG (20 mM) for 30 or 60 min, and then the nuclear and cytosolic fractions were harvested, and the protein expression levels of p65 in the cytoplasm or nucleus were analyzed by Western blotting. **(B)** BV2 cells were treated with R-2HG (20 mM) for 0, 30, and 60 min, and then the cells were stained with anti-p65 (green) antibody. Nuclei were stained with DAPI (blue) to determine the nuclear localization of p65; the staining results were observed by confocal microscopy. Scale bar, 20 mm. **(C)** BV2 cells were treated with R-2HG (20 mM) for 0, 15, 30, and 60 min, and protein expression levels of p65, p-p65, IκBα, and p-IκBα were detected by Western blotting. β−Actin was used as an internal control for the whole-cell lysate or cytosolic fractions. Levels of nuclear proteins were quantified using densitometry and normalized to Histone H3. All results are presented as the means ± SD (n = 3) and are representative of three independent experiments. Student’s t-test was used for unpaired comparisons of two groups. Differences between more than two groups were determined by one-way ANOVA or two-way ANOVA with a Tukey’s *post-hoc* test. **p < 0.01, ***p < 0.001, and ****p < 0.0001.

Next, we checked the nuclear translocation of NF-κB subunit p65 to explore how R-2HG inhibited microglial activation via the NF-κB pathway. BV2 cells were stimulated with CM from GL261 or ALTS1C1 cells, and then the separation of the nuclear and cytoplasmic fraction was performed. As the results showed, the nuclear translocation of p65 was significantly increased under the stimulation of CM, and R-2HG treatment markedly reduced p65 nuclear translocation (**Figure 3A**, **Supplementary Figure S6A**). Furthermore, immunofluorescence analysis also showed that p65 (green) was mainly located in the cytoplasm under normal conditions and translocated to the nucleus after CM treatment for 30 or 60 min, whereas R-2HG significantly prevented this nuclear translocation (**Figure 3B**, **Supplementary Figure S6B**).

Previous studies show that the phosphorylation of p65 or IκBα regulates the nuclear translocation of p65 (27, 28), so we examined their protein and phosphorylation levels after R-2HG treatment. We found that R-2HG had no effect on the protein levels of p65 and IκBα but reduced their phosphorylation levels (**Figure 3C**), which was responsible for p65 activation. Therefore, these data suggested that R-2HG attenuated the CM-induced activation of the NF-κB pathway in BV2 cells.

### 3.3 R-2HG downregulates IL-6 expression by inhibiting α-KG-dependent enzymes

**Figure 4:**
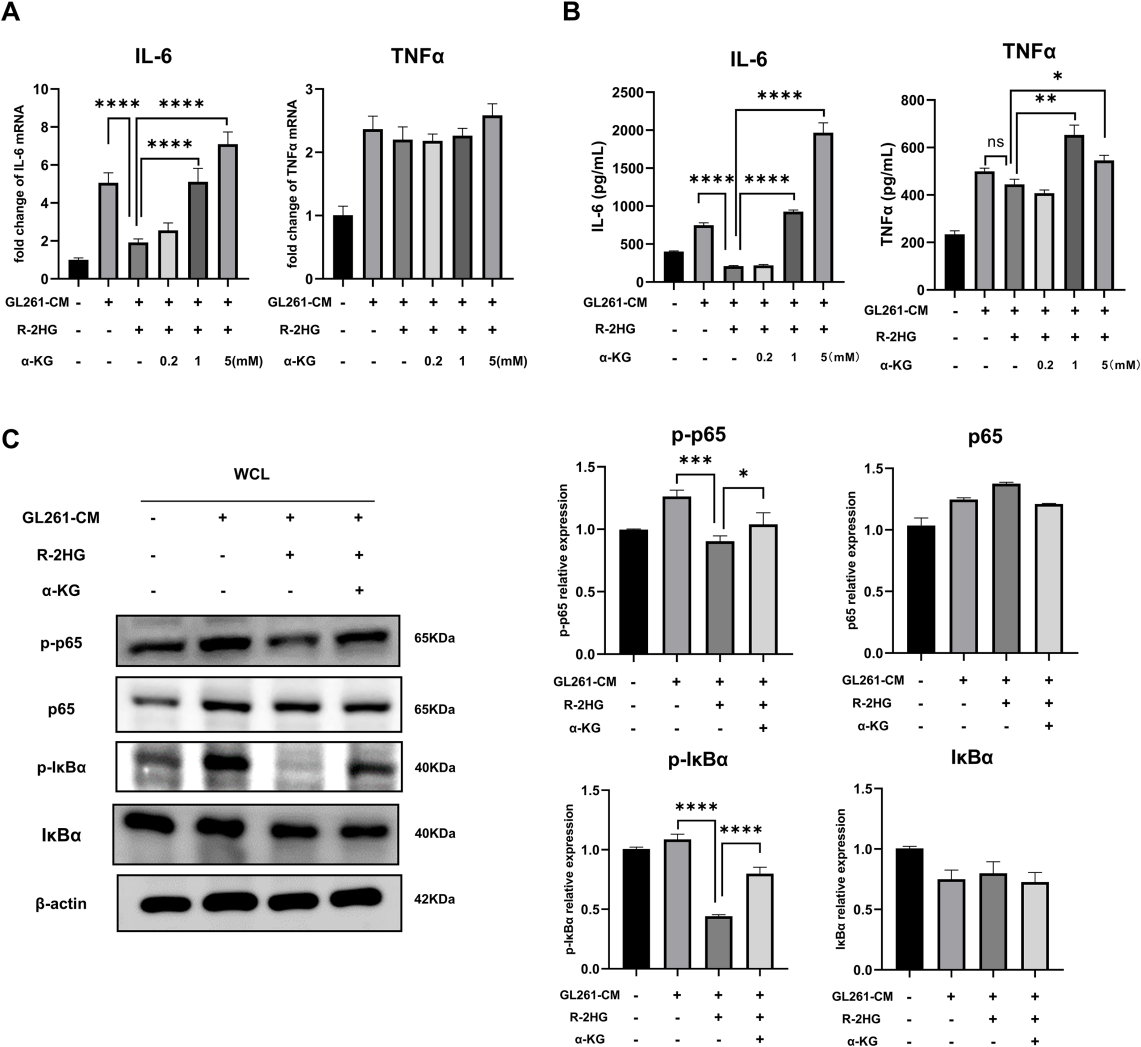
The addition of α-KG reverses the inhibitory effect of R-2HG on IL-6 in GL261-CM-induced BV2 cells. In the conditioned medium stimulation system for GL261 glioma cells, R-2HG-treated BV2 cells were added with 0.2, 1, and 5 mM of dimethyl-2-hydroxyglutarate (dimethyl-2-oxoglutarate, α-KG), respectively. **(A)** After incubation for 6 h, the cells were assayed by qPCR for IL-6 and TNFα mRNA expression levels. **(B)** After incubation for 24 h, the protein expression levels of IL-6 and TNFα in cell supernatants were determined by ELISA. **(C)** Stimulated with conditioned medium from GL261 glioma cells, R-2HG-treated BV2 cells were added with 1 mM α-KG for 30 min; the protein expression levels of p65, p-p65, IκBα, and p-IκBα were detected by Western blotting. β-Actin was used as the internal control. All results are presented as the means ± SD (n = 3). Differences between more than two groups were determined by one-way ANOVA with a Tukey’s *post-hoc* test. *p < 0.05, **p < 0.01, ***p < 0.001, and ****p < 0.0001.

As R-2HG can act as a competitive inhibitor for a range of α-KG-dependent enzymes (7) and these enzymes regulate histone, DNA, and RNA methylation, as well as macrophage activation (29–31), we supposed that R-2HG might influence the NF-κB pathway by competing with α-KG. Thus, the inhibition of IL-6 by R-2HG should be reversed by the addition of α-KG. We added different concentrations of α-KG back to the R-2HG-treated BV2 cells. The results showed that the addition of α-KG could indeed rescue the mRNA and protein expression of IL-6 in R-2HG- and CM-treated BV2 cells. Although α-KG increased the protein level of TNFα, its RNA level was not affected, and R-2HG did not suppress the expression of TNFα (**Figures 4A, B**, **Supplementary Figure S7A**). Furthermore, the addition of α-KG recovered the phosphorylation levels of p65 and IκBα, which was attenuated by R-2HG (**Figure 4C**). These results suggested that R-2HG inhibited IL-6 transcription in BV2 cells by competing with α-KG, indicating that this effect might rely on α-KG-dependent enzymes.

### 3.4 R-2HG downregulates IL-6 expression by inhibiting FTO in BV2 cells

**Figure 5:**
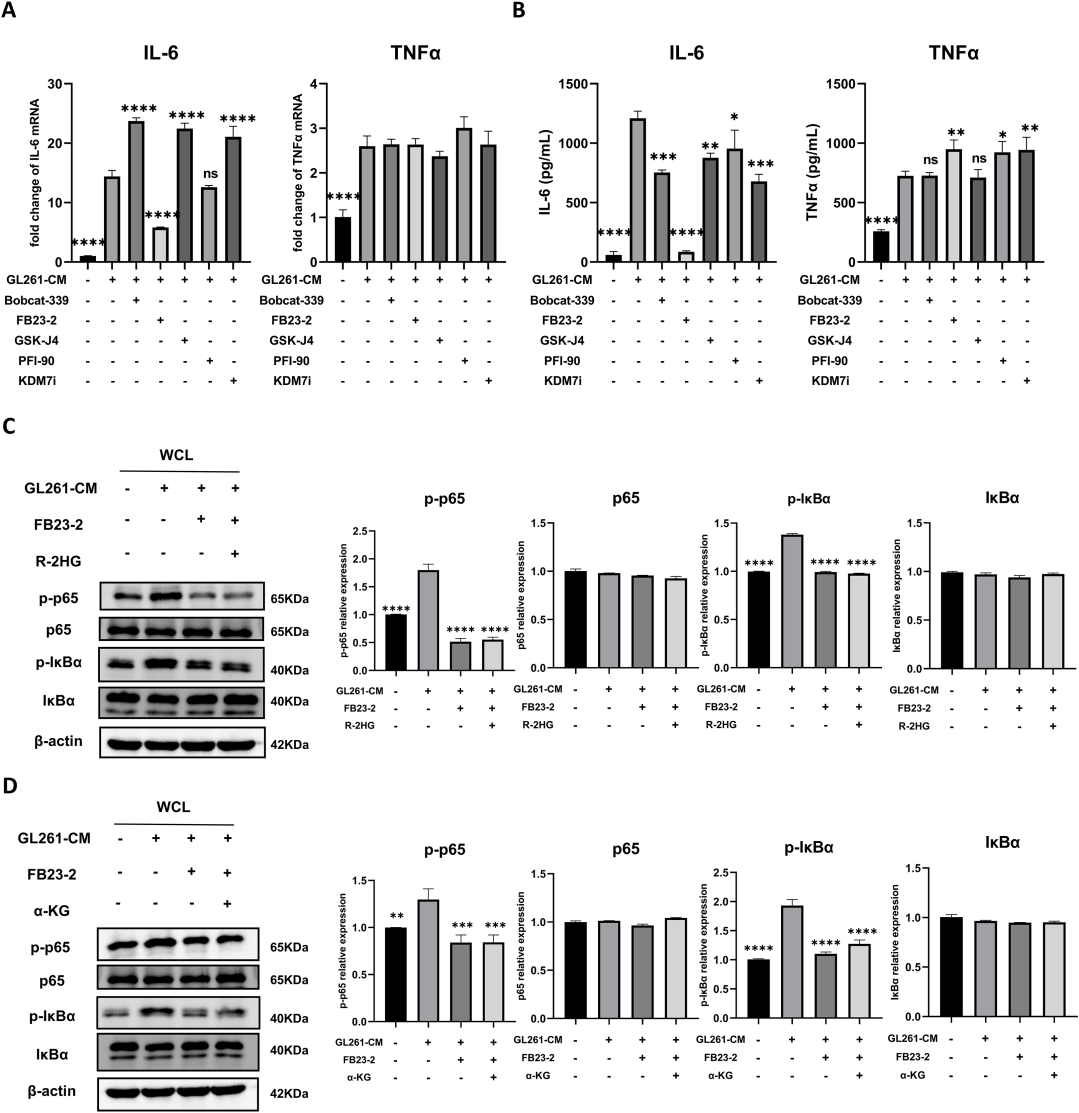
R-2HG suppresses NF-κB pathway activation by inhibiting FTO. In the conditioned medium from GL261 glioma cells, **(A)** BV2 cells were treated with Bobcat-339 (TET1/2 inhibitor, 50 μM), FB23-2 (FTO inhibitor, 5 μM), GSK-J4 (KDM6 inhibitor, 5 μM), PFI-90 (KDM3 inhibitor, 5 μM), and KDM7 inhibitor (20 μM) for 6 h. The mRNA levels of IL-6 and TNFα in BV2 cells were determined using qPCR. **(B)** BV2 cells were treated with Bobcat-339 (50 μM), FB23-2 (5 μM), GSK-J4 (5 μM), PFI-90 (5 μM), and KDM7 inhibitor (20 μM) for 24 h, respectively. The protein levels of IL-6 and TNFα in BV2 cells were determined using ELISA. **(C)** BV2 cells were treated with FB23-2 (5 μM) or cotreated with R-2HG (20 mM) and FB23-2 (5 μM) for 1 h, and then the protein expression levels of p65, p-p65, IκBα, and p-IκBα were detected by Western blotting. **(D)** BV2 cells were treated with FB23-2 (5 μM) or cotreated with α-KG (1 mM) and FB23-2 (5 μM) for 1 h, and then the protein expression levels of p65, p-p65, IκBα, and p-IκBα were detected by Western blotting. β-Actin was used as the internal control. All results are presented as the means ± SD (n = 3) and are representative of three independent experiments. Differences between more than two groups were determined by one-way ANOVA with a Dunnett’s *post-hoc* test. *p < 0.05, **p < 0.01, ***p < 0.001, and ****p < 0.0001 compared with CM. "ns" indicates a non-significant result (p ≥ 0.05).

The α-KG-dependent enzymes, including JmjC-family histone demethylases, TET-family DNA demethylases, and RNA demethylases such as FTO, play critical roles in histone, DNA, or RNA methylation modification (7). Recent studies showed α-KG-dependent enzymes involved in inflammatory response. Itaconate, an analog of α-KG, could impair the inflammatory response in macrophages by directly binding with TET2 (30). Moreover, the knockdown of FTO inhibits the NF-κB signaling pathway, leading to the suppression of macrophage polarization (31). As R-2HG inhibited IL-6 transcription in BV2 which relies on α-KG-dependent enzymes, we employed the inhibitors for some typical α-KG-dependent enzymes, including TET1/2, FTO, and demethylase targeting H3K9, H3K27, and H4K20, which are associated with transcriptional repression (32). It was shown that only the FTO inhibitor markedly reduced the mRNA and protein expression of IL-6 in GL261 CM-stimulated BV2 cells with minor increased protein expression of TNFα (**Figure 5A, B**). Furthermore, the FTO inhibitor decreased IL-6 expression in BV2 cells induced by CM from GL261 in a dose-dependent manner, indicating the relationship between FTO and IL-6 expression (**Supplementary Figures S8A-B**). The FTO inhibitor also inhibited the phosphorylation of p65 and IκBα, while combining with R-2HG did not show any further inhibitory effects (**Figure 5C**). The addition of α-KG was unable to rescue the phosphorylation of p65 and IκBα under FTO inhibitor treatment, suggesting that R-2HG inhibits IL-6 activation via FTO activity (**Figure 5D**).

**Figure 6:**
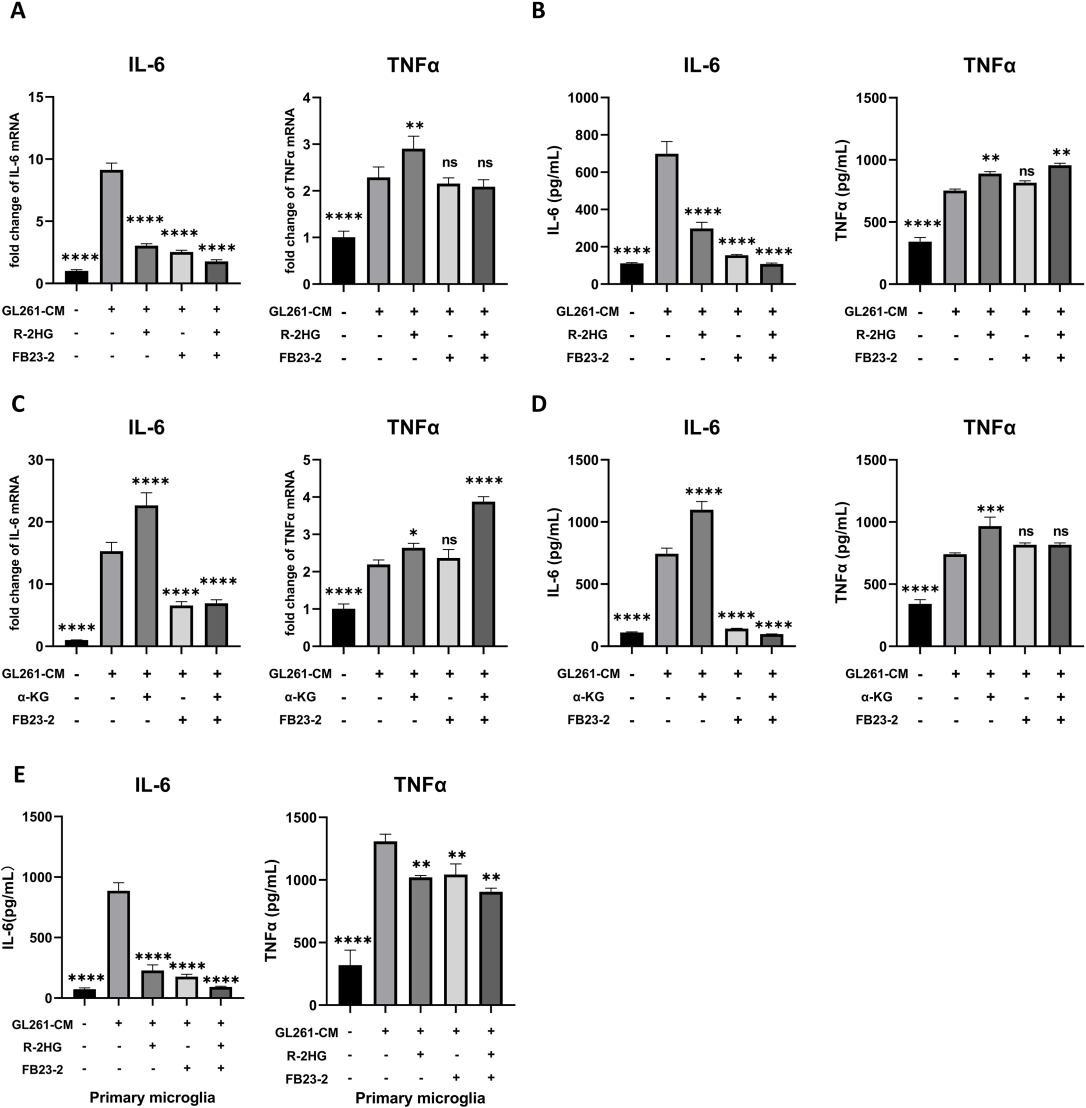
R-2HG specifically downregulates IL-6 expression by inhibiting FTO in BV2 cells. **(A)** Stimulated with conditioned medium from GL261 glioma cells, BV2 cells were treated with R-2HG (20mM), FB23-2 (5µM) or co-treated with R-2HG (20mM) and FB23-2 (5µM), respectively. After incubation for 6 h, the mRNA levels of IL-6 and TNFα were analyzed by qPCR. **(B)** After incubation for 24 h, the protein levels of IL-6 and TNFα in supernatants were determined by ELISA. **(C)** Stimulated with conditioned medium from GL261 glioma cells, primary microglia were treated with R-2HG (20mM), FB23-2 (5µM) or co-treated with R-2HG (20mM) and FB23-2 (5µM), respectively. After incubation for 24 h, the protein levels of IL-6 and TNFα in supernatants were determined by ELISA. **(D)** BV2 cells were treated with α-KG (1mM), FB23-2 (5µM) or co-treated with α-KG (1mM) and FB23-2 (5µM), respectively. After incubation for 6 h, the mRNA levels of IL-6 and TNFα were analyzed by qPCR. **(E)** And after incubation for 24 h, the protein levels of IL-6 and TNFα in supernatants were determined by ELISA. All results are presented as the means ± SD (n = 3) and are representative of three independent experiments. Differences between more than two groups were determined by one-way ANOVA with a Dunnett’s *post-hoc* test. *p < 0.05, **p < 0.01, ***p < 0.001, and ****p < 0.0001 compared with CM. "ns" indicates a non-significant result (p ≥ 0.05).

Furthermore, the combination of the FTO inhibitor and R-2HG did not produce any additional inhibitory effect on IL-6 compared with FTO inhibitor treatment alone, as both significantly reduced IL-6 expression at the mRNA and protein levels (**Figure 6A, B**). A similar trend was observed in primary microglia (**Figure 6C**). Additionally, supplementation with α-KG failed to rescue IL-6 expression (**Figure 6D, E**). Collectively, these findings indicated that R-2HG attenuated microglial activation by competing with α-KG to inhibit FTO activity, leading to the specific downregulation of IL-6 expression.

## 4 Discussion

In this study, we investigated the effect of the oncometabolite R-2HG on microglial activation induced by glioma conditioned medium (CM). We found that CM from GL261 or ALTS1C1 mouse glioma cells significantly induced BV2 cells and primary microglial activation by increasing the expression of cytokines such as IL-6 and TNFα. R-2HG treatment inhibited the production of pro-inflammatory cytokine and chemokine production such as IL-6, CCL2, and CXCL10, whereas it had no inhibitory effect on TNFα, IL-1β, and iNOS. Mechanistically, IL-6 is transcribed by NF-κB, and R-2HG exerts an inhibitory effect on CM-induced activation of the NF-κB signaling pathway by competitively inhibiting the activity of the α-KG-dependent demethylase FTO.

The discovery of mutations in genes encoding key metabolic enzymes has highlighted the direct link between metabolic alterations and diseases. In particular, the mutations in fumarate hydratase, succinate dehydrogenase, and isocitrate dehydrogenase (enzymes in the tricarboxylic acid cycle) revealed how mitochondrial metabolites accumulate and act as oncometabolites (33). In addition to established intracellular functions, it has been observed that oncometabolites have non-autonomous effects on tumor cells and mediate intercellular crosstalk in the tumor microenvironment (33). For example, the cancer cell-derived fumarate could impair the antitumor ability of CD8^+^ T cells in the tumor microenvironment (34). In addition, in macrophages, the metabolites of succinate and itaconate have received much more attention. Cancer cells were reported to release succinate into their microenvironment and activate succinate receptor (SUCNR1) signaling to polarize macrophages into TAM, promoting cancer cell migration, invasion, and metastasis (35). Itaconate is significantly upregulated in macrophages from mice with tumor burden and acts as a key regulator of macrophage metabolism, ROS production, and tumor progression (36).

The oncometabolite R-2HG is produced by IDH1 or IDH2 mutations in tumor cells and accumulated in the tumor microenvironment to levels up to 30 mM (17). R-2HG is an inhibitor of a variety of α-ketoglutarate (α-KG)-dependent dioxygenases and leads to alterations in the epigenetics, resulting in oncogenic transformation (37). Previous studies reported that R-2HG could dampen the inflammatory responses, which is in part independent on α-KG-dependent dioxygenases or epigenetic remodeling (38). In addition, IDH1 mutant gliomas exhibit an “immune-cold” microenvironment with abundant microglia, moderate myeloid cells, minimal B cells, and T cells, showing less immunoreaction than IDH wild-type (WT) counterparts (18, 19). To model this differential immune response, we stimulated microglia with conditioned medium (CM) from IDH-wild-type glioma cells, which provides a pro-inflammatory “tumor signal” lacking R-2HG. We then added R-2HG to this system to simulate the specific paracrine influence of an IDH-mutant tumor. Moreover, we found that R-2HG regulates the glioma microenvironment by decreasing IL-6, CCL2, CXCL10, and MMP-14 in microglia. Notably, R-2HG disodium salt specifically downregulated the expression of IL-6 in microglia, with no effect on TNFα and IL-1β, which is similar to itaconate, selective inhibiting IL-6, without affecting TNFα (39).

Microglia are the major immune cell population in the glioma microenvironment and express a variety of receptors on the surface, including toll-like receptors (TLRs), which recognize invading pathogens and endogenous harmful stimulation to induce innate and adaptive immune responses (40). A well-characterized pathway for such activation, exemplified by the responses to LPS, is the NF-κB signaling pathway (41). Our results indicate that, similar to LPS, conditioned medium from GL261 or ALTS1C1 glioma cells also robustly activates this canonical NF-κB pathway in microglia, as shown by the phosphorylation of IκBα and p65, and the subsequent nuclear translocation of p65. While the specific receptor(s) initiating this response to our CM remains to be identified, the downstream activation of NF-κB is clear. Additionally, R-2HG does not affect the protein expression of p65 but inhibits p65 activation and nuclear translocation. Consequently, the inhibitory effect of R-2HG—which acts by preventing IκBα and p65 phosphorylation and blocking p65 nuclear translocation—highlights a potent mechanism for suppressing microglial inflammatory activation within the glioma niche. Interestingly, previous studies have reported that, in bone marrow stromal cells, R-2HG could induce NF-κB activation in an IκB kinase-independent manner, which is helpful for the establishment of a supportive bone marrow stromal niche to promote acute myeloid leukemia (AML) progression (42). This suggests that R-2HG may have a differential effect for immune cells, stromal cells, or other cells in the tumor microenvironment, which depends on the cell types.

We also found that the addition of α-KG could rescue the IL-6 expression in R-2HG and CM-treated BV2 cells, as well as the phosphorylation levels of p65 and IκBα, which indicates that this effect might relay on α-KG-dependent enzymes. Therefore, we employed the inhibitors for some typical α-KG-dependent enzymes; only the FTO inhibitor significantly reduced the expression of IL-6 in GL261 CM-stimulated BV2 cells. FTO is a member of the Alkb family catalyzing the demethylation of a wide range of substrates in a α-KG-dependent manner, such as m6A and m6Am in mRNAs, m6A and m6Am in snRNAs, and m1A in tRNAs (43), which is involved in multiple mRNA-related processes, including transcriptional stability, alternative splicing, mRNA translocation, and protein translation (44). An increasing number of studies have reported that FTO-mediated epigenetic modifications play an important role in the regulation of immune responses and metabolic diseases, such as acute myeloid leukemia, heart failure, and type 2 diabetes (45–47). FTO affects macrophage foam cell transformation, a critical step in the initiation and progression of atherosclerosis, primarily by activating AMPK-induced cholesterol flux and inhibiting PPAR-γ-mediated lipid uptake (48). More importantly, previous studies have shown that FTO is involved in multiple inflammatory disorders. For example, it is reported that FTO overexpression significantly enhanced inflammatory responses in the human cardiomyocyte AC16 cell line (49) or RAW264.7 cells and bone marrow-derived macrophages (50). FTO knockdown inactivates NF-κB and decreases the mRNA stability of STAT1 and PPAR-γ via YTHDF2 to inhibit macrophage activation (31). Furthermore, knockdown of FTO inhibited NF-κB activation and osteoclast-specific gene expression in an osteoporosis mode (51). Consistently, our study also found that R-2HG specifically downregulates IL-6 expression by inhibiting FTO to prevent nucleus translocation of p65, inhibiting inflammatory activation in microglia. Interestingly, the FTO inhibitor slightly increased TNFα protein expression without affecting its RNA level. Furthermore, the addition of α-KG rescued the R-2HG-mediated downregulation of the NF-κB/IL-6 pathway. However, α-KG could not reverse the suppression of p-p65 and IL-6 induced by the FTO inhibitor. This specific inability to rescue the effects of the FTO inhibitor further confirms that FTO is the primary target of R-2HG. In addition to its function as a RNA demethylase, FTO also oxidatively demethylates 3mT in ssDNA, with negligible activity on 3mT in dsDNA (43). Thus, the regulatory effect of FTO on specific target genes of NF-κB may be related to its DNA demethylase activity. Nevertheless, the specific methylation sites where FTO acts remains to be identified.

## 5 Conclusions

In conclusion, our results suggest that R-2HG inhibits FTO activity and reduces phosphorylation of NF-κB p65 and IκBα by competing with α-KG, subsequently inhibiting microglial activation and impairing antitumor function of microglia. Our study highlights that R-2HG not only is an oncometabolite but also serves as a signal that regulates the tumor microenvironment in order to modulate immune cells and their cytokines, thus providing new mechanistic insights into the role of IDH mutations in glioma development.

## Data Availability

Publicly available datasets were analyzed in this study. This data can be found here: http://www.proteinatlas.org.
