# Supplementary material for: The oncometabolite R-2-hydroxyglutarate inhibits microglial activation via the FTO/NF-κB pathway
Source: Front Oncol. 2025 Nov 24;15:1525761. doi: 10.3389/fonc.2025.1525761 (PMC12683333; doi:10.3389/fonc.2025.1525761)
Supplement: Supplementary file 1 [file DataSheet1.docx]

Supplementary Material

# Supplementary Figures and Tables

## Supplementary Tables

**Supplementary Table 1.** **DNA sequences of the primers used for qPCR**

| Primer | Sequence |
| --- | --- |
| IL-6 | CTACCCCAATTTCCAATGCT  ACCACAGTGAGGAATGTCCA |
| CCL-2 | TTAAAAACCTGGATCGGAACCAA  GCATTAGCTTCAGATTTACGGGT |
| CXCL-10 | CCAAGTGCTGCCGTCATTTTC  GGCTCGCAGGGATGATTTCAA |
| MMP-14 | CAGTATGGCTACCTACCTCCAG  GCCTTGCCTGTCACTTGTAAA |
| TNFα | CTATGACCGGCATCTGTGGAA  AGCAACCTTGTCCAACCCTTG |
| IL-1β | ATCTTTTGGGGTCCGTCAACT CTACCCCAATTTCCAATGCT |
| iNOS | CCAAGCCCTCACCTACTTCC CTCTGAGGGCTGACACAAGG |
| β-actin | TCCATCATGAAGTGTGACGT TACTCCTGCTTGCTGATCCAC |

## Supplementary Figures


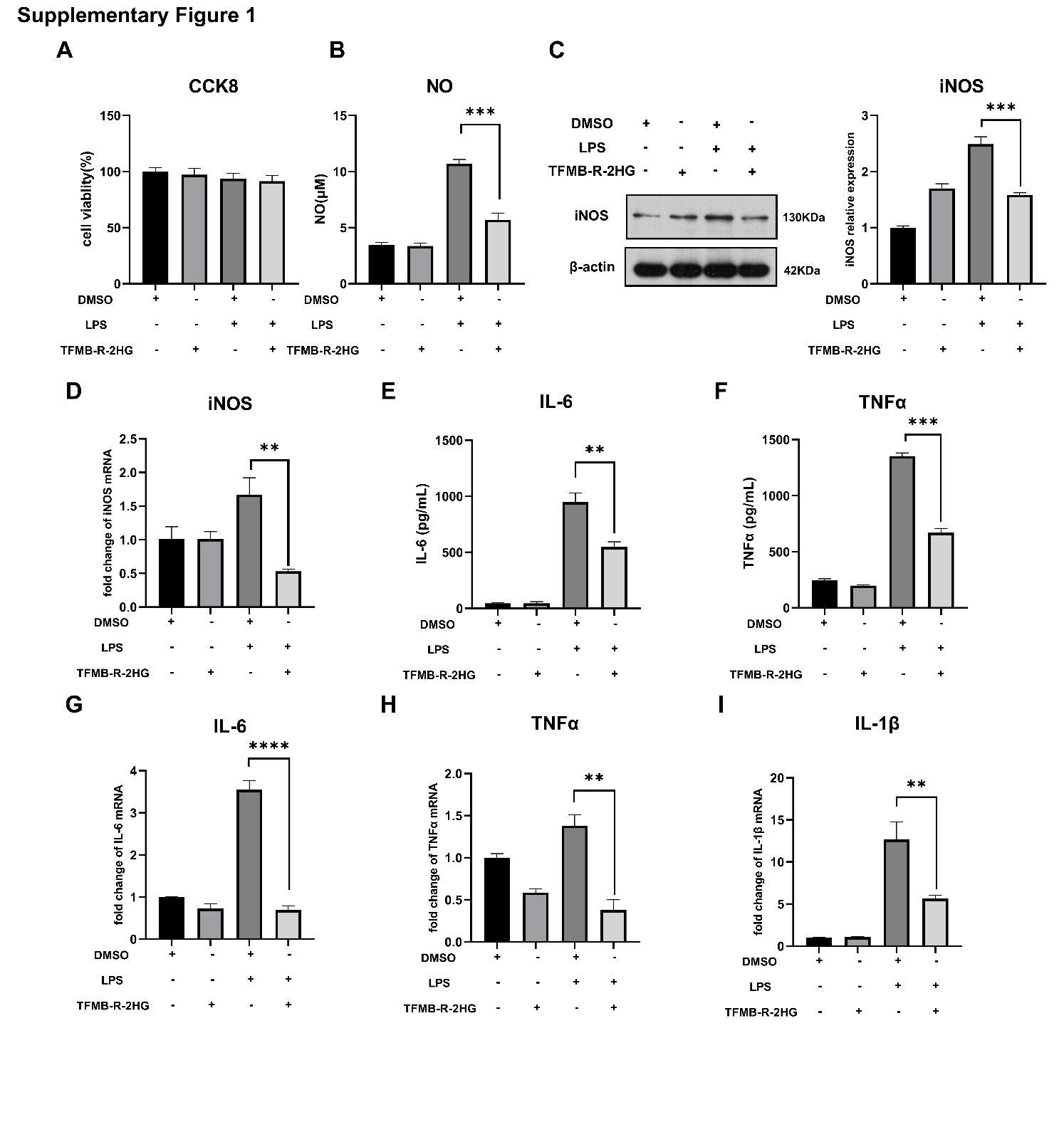


**Supplementary Figure 1.** **TFMB-R-2HG inhibited the inflammatory activation of BV2 cells stimulated by LPS.** BV2 cells were treated with 1mg/mL of LPS and 1mM of TFMB-R-2HG, while the control group was added an equal volume of DMSO. (A) After incubation for 24h, the cell viability of each group was detected using the CCK-8 assay. (B) The cell supernatants were collected after 24h and the NO content in the supernatants was measured. (C) The level of iNOS was analyzed using Western blotting after treatment for 24h. β-actin was used as the internal control. (D) After incubation for 6h, the mRNA expression levels of iNOS were determined using qPCR. (E-F) After incubation for 24h, the levels of secreted IL-6 and TNFα in the supernatants of BV2 cells were measured using ELISA. (G-I) After incubation for 6h, The mRNA expression levels of specific genes were determined using qPCR. All results are presented as the means ± SD (n=3) and are representative of three independent experiments. Student’s t-test was used for unpaired comparisons of two groups. Differences between more than two groups were determined by one-way ANOVA with a Tukey’s post hoc test. ***p* < 0.01, ****p* < 0.001 and *****p* < 0.0001.


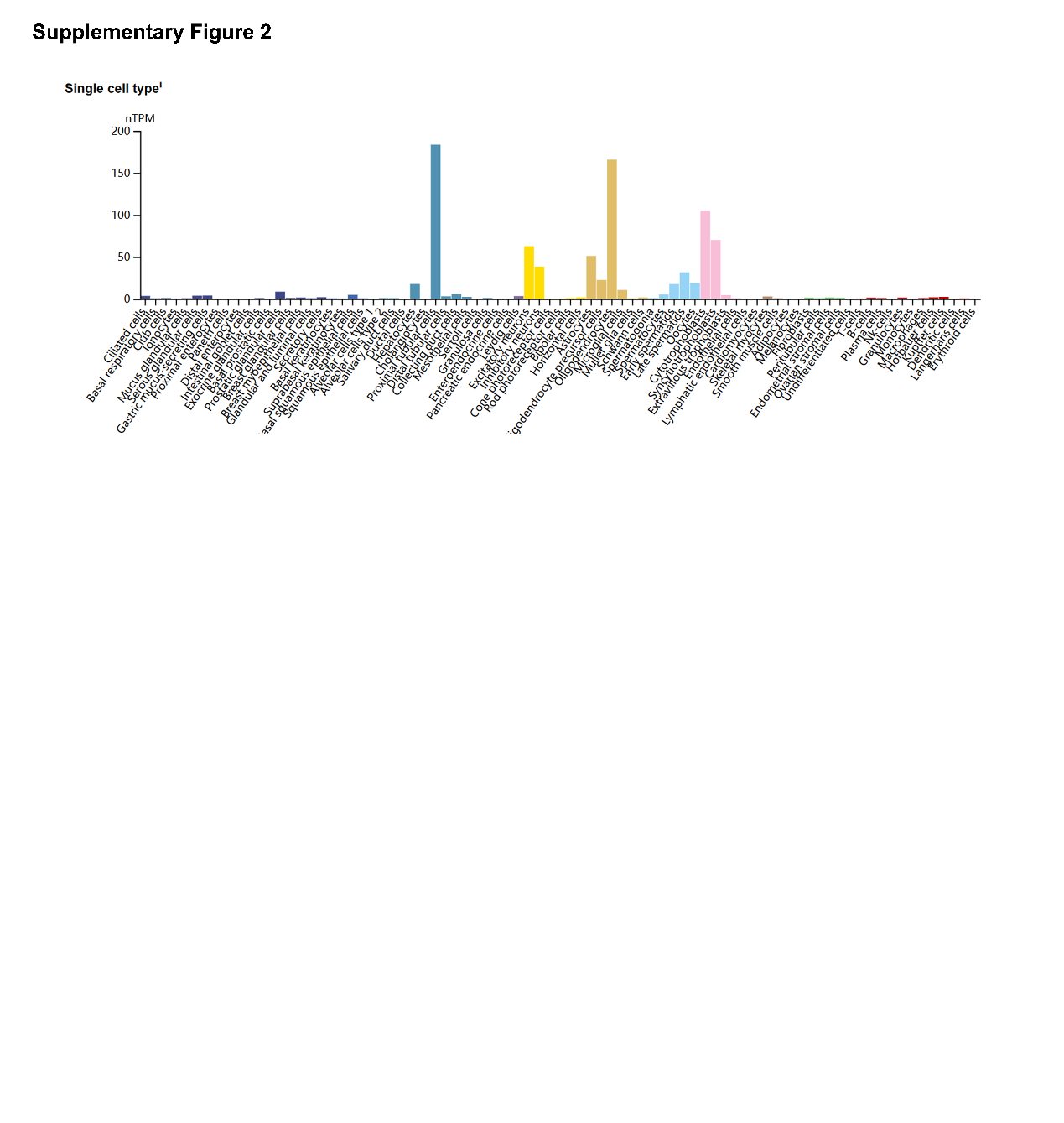


**Supplementary Figure 2.** **R-2HG transporter protein SLC13A3 is highly expressed in glial cells.** The transporter protein SLC13A3, which is responsible for transporting R-2HG, is highly expressed in glial cells such as astrocytes and microglia. (The data is from <http://www.proteinatlas.org>)


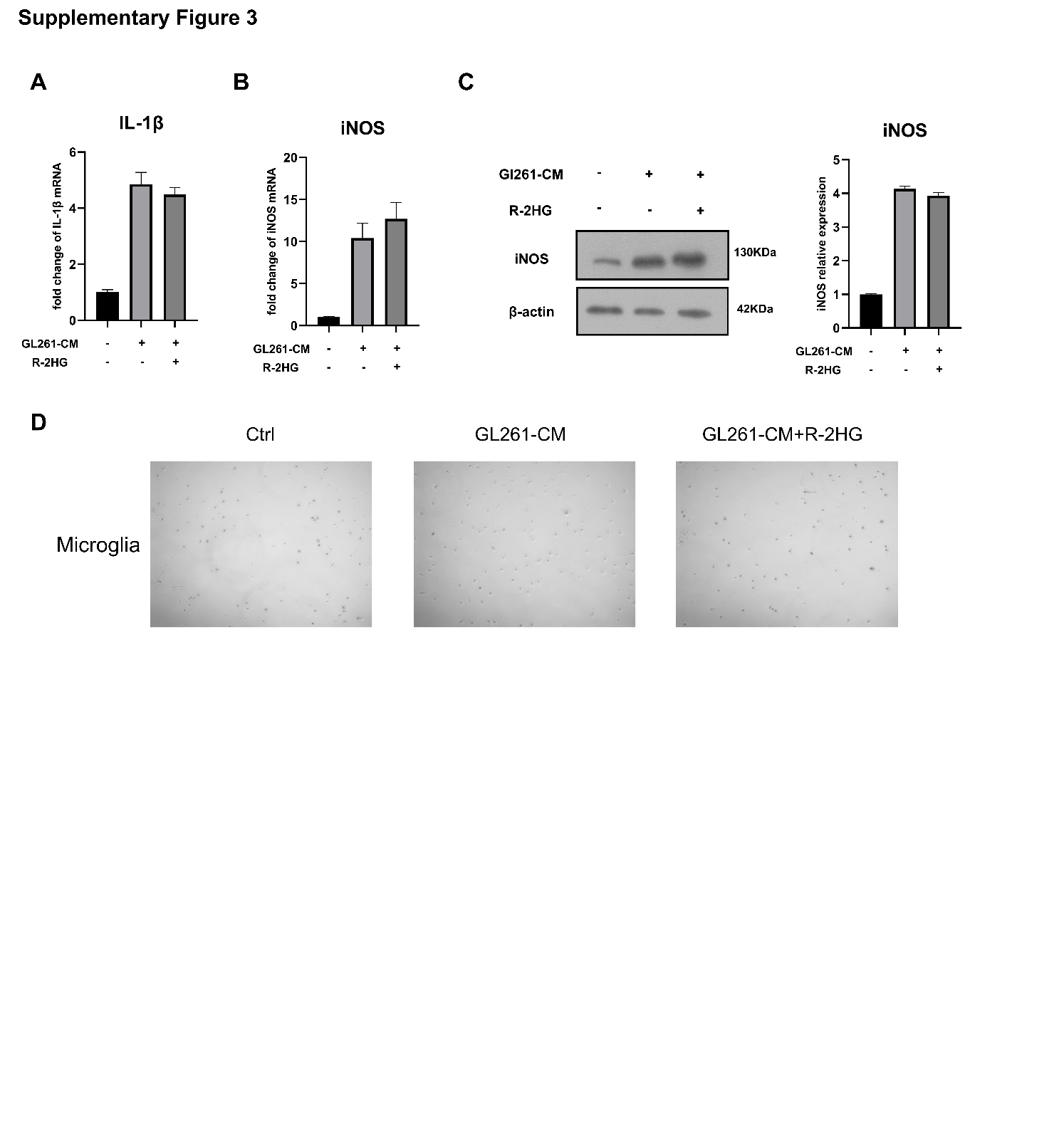


**Supplementary Figure 3.** **The effects of R-2HG on IL-1β and iNOS in BV2 cells and on the morphology of primary microglia.** (A-B) BV2 cells were treated with conditioned medium from GL261 glioma cells and 20mM R-2HG, after incubation for 6h, the mRNA expression levels of IL-1β and iNOS were determined using qPCR. (C) The level of iNOS was analyzed using Western blotting after treatment for 24h. β-actin was used as the internal control. All results are presented as the means ± SD (n = 3) and are representative of three independent experiments. Student’s t-test was used for unpaired comparisons of two groups. ***p* < 0.01, ****p* < 0.001 and *****p* < 0.0001 compared to CM.

**
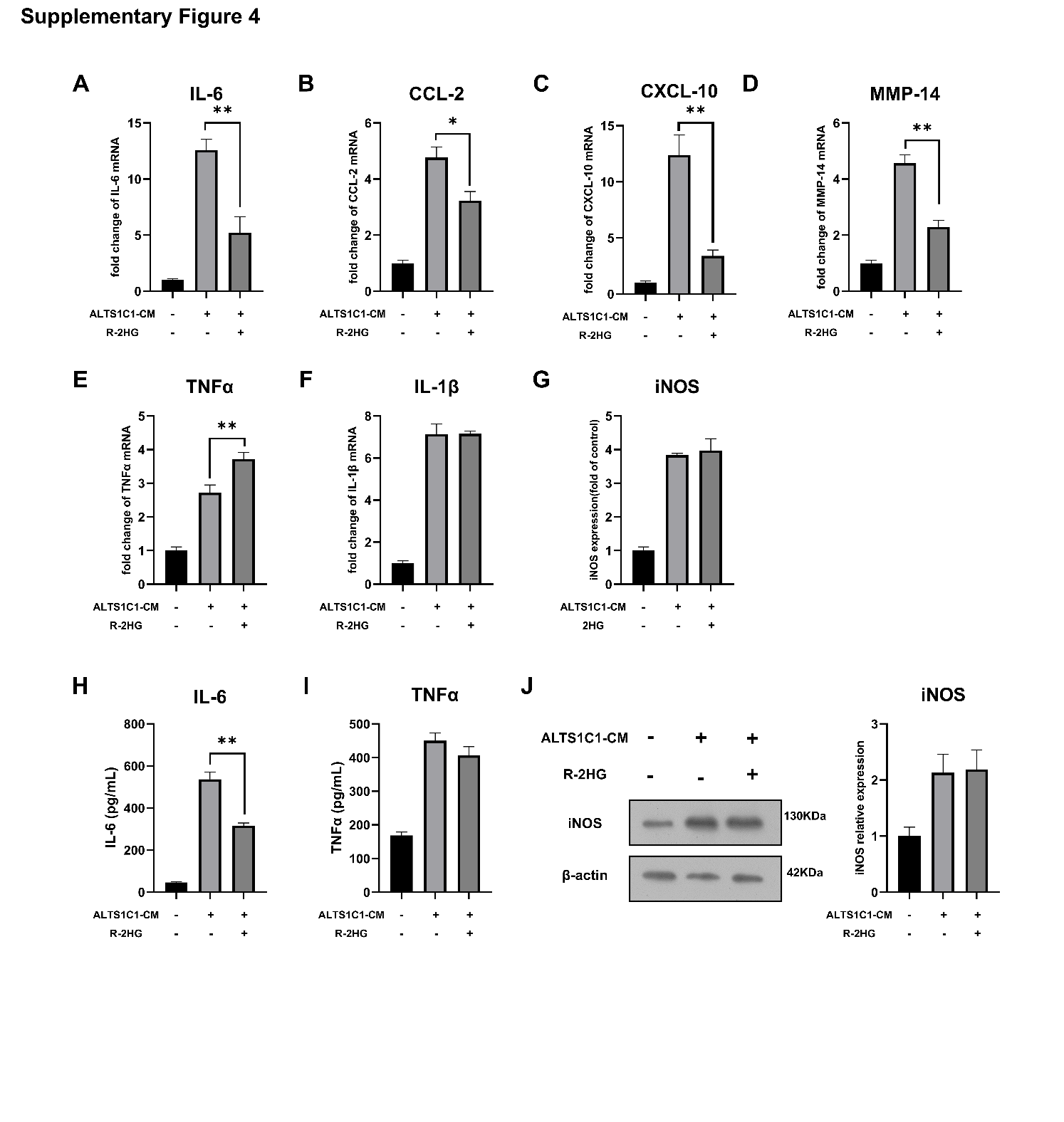
**

**Supplementary Figure 4.** **R-2HG dampens ALTS1C1-CM-induced inflammatory activation in the BV2 cell.** BV2 cells were treated with conditioned medium from ALTS1C1 glioma cells and 20mM R-2HG. (A-G) After incubation for 6h, The mRNA expression levels of specific genes were determined using qPCR. (H-I) After incubation for 24h, the levels of secreted IL-6 and TNFα in the supernatants of BV2 cells were measured using ELISA. (J) The level of iNOS was analyzed using Western blotting after treatment for 24h. β-actin was used as the internal control. All results are presented as the means ± SD (n = 3) and are representative of three independent experiments. Student’s t-test was used for unpaired comparisons of two groups. ** *p* < 0.01, ****p* < 0.001 and *****p* < 0.0001 compared to CM.


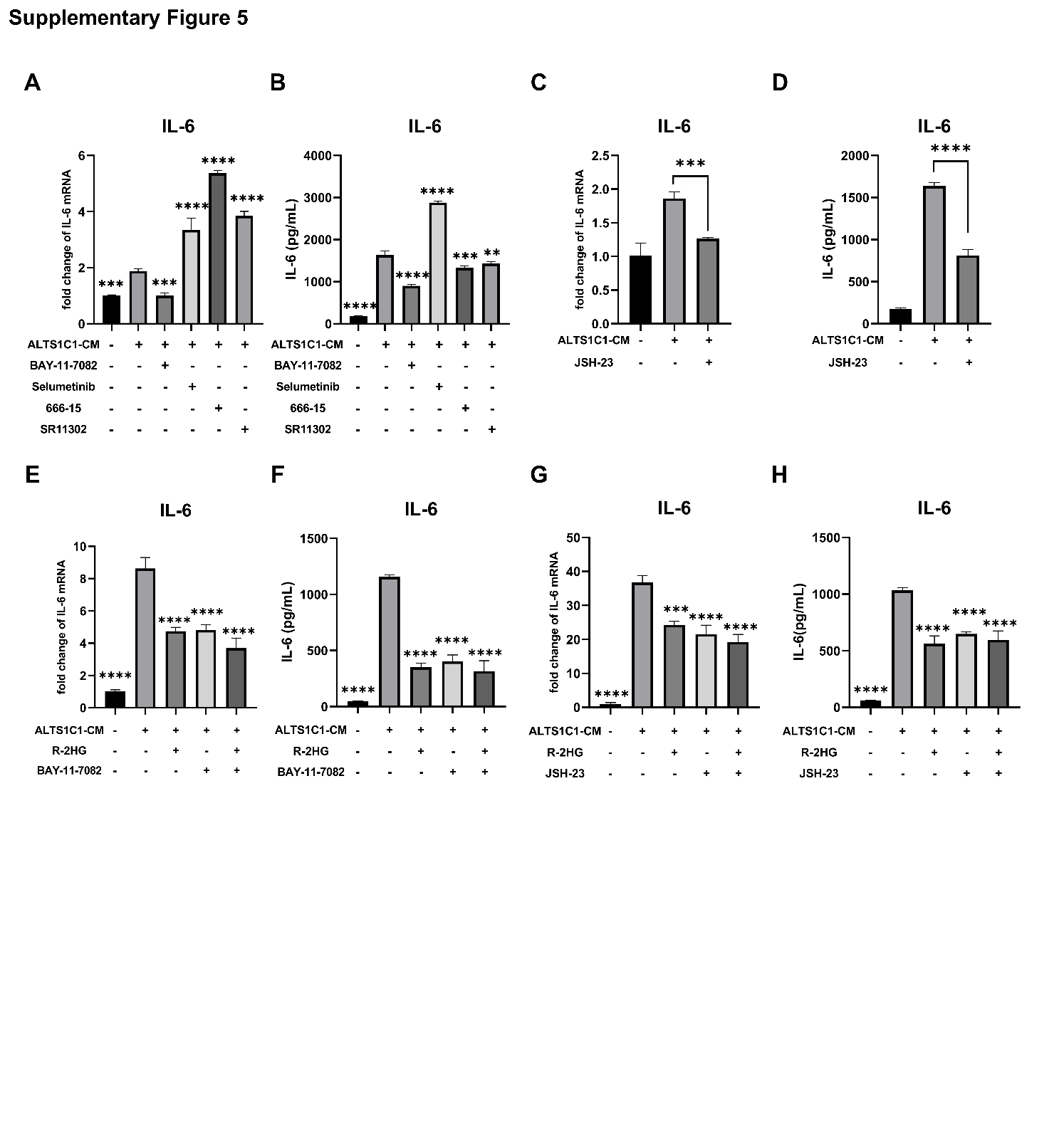


**Supplementary Figure 5.** **R-2HG attenuates IL-6 production by inhibiting the NF-κB pathway in ALTS1C1-CM induced BV2 cells.** (A-B) Stimulated with conditioned medium from ALTS1C1 glioma cells, BV2 cells were treated with inhibitors of different transcription factors of IL-6, and the cell pellets and cell supernatants were analyzed for mRNA and protein expression levels of IL-6 using qPCR and ELISA, respectively. (C-D) BV2 cells were treated with another NF-κB inhibitor, JSH-23, under the stimulation of conditioned medium from ALTS1C1 glioma cells, the cell pellets and cell supernatants were assayed for IL-6 mRNA and protein expression levels using qPCR and ELISA, respectively. (E-F) BV2 cells were treated with R-2HG, BAY11-7082, or co-treated with R-2HG and BAY11-7082, respectively, then the mRNA and protein expression levels of IL-6 were examined. (G-H) BV2 cells were treated with R-2HG, JSH-23, or co-treated with R-2HG and JSH-23, respectively, and the mRNA and protein expression levels of IL-6 were examined. All results are presented as the means ± SD (n = 3) and are representative of three independent experiments. Student’s t-test was used for unpaired comparisons of two groups. Differences between more than two groups were determined by one-way ANOVA with a Dunnett’s post hoc test. ***p* < 0.01, ****p* < 0.001 and *****p* < 0.0001 compared to CM.


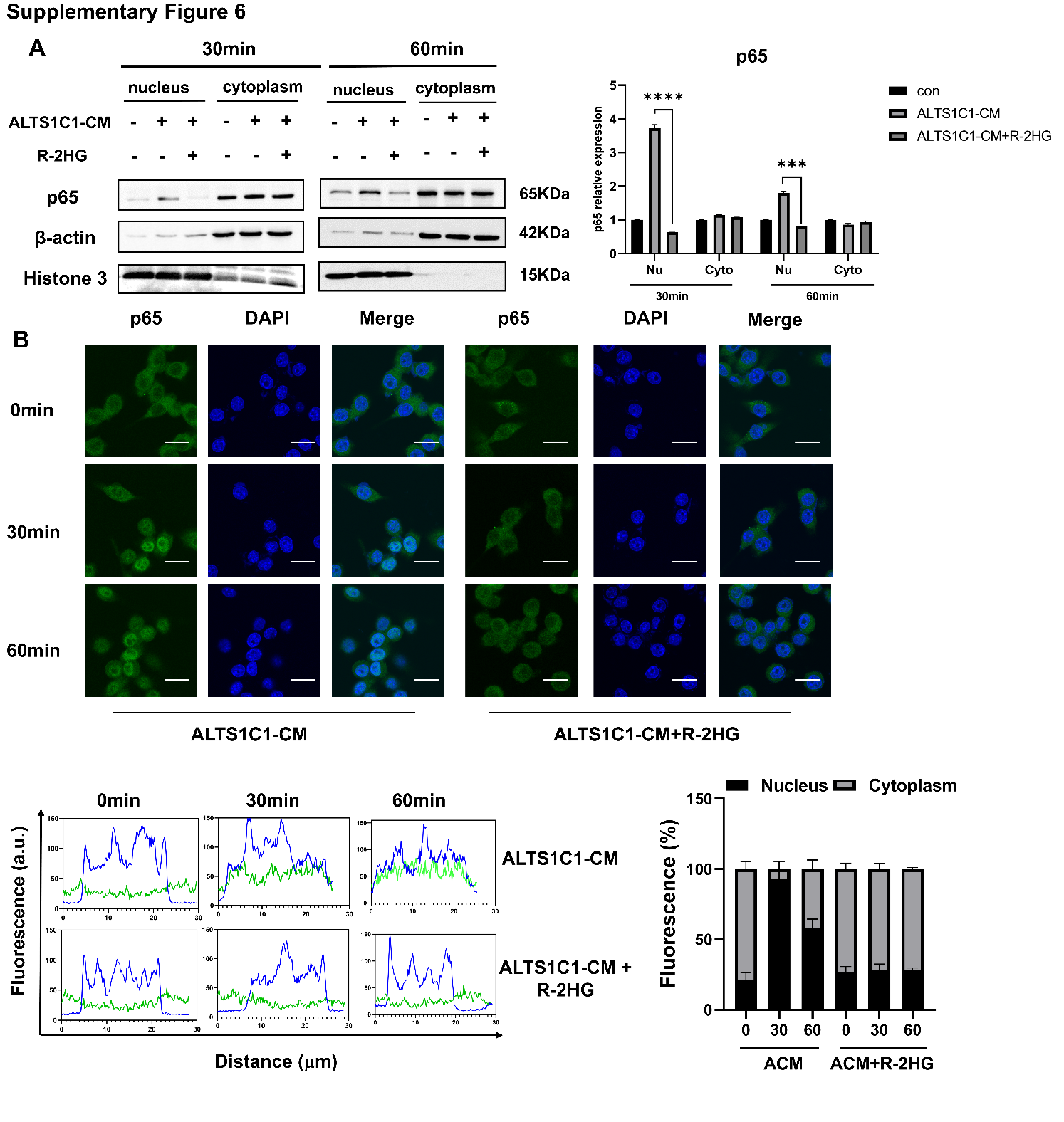


**Supplementary Figure 6.** **R-2HG reduces p65 phosphorylation and nuclear entry in ALTS1C1-CM induced BV2 cells.** Stimulated with conditioned medium from ALTS1C1 glioma cells, BV2 cells were treated with R-2HG for 30 and 60min. (A) The nuclear and cytosolic fractions were harvested, and the protein expression levels of p65 in the cytoplasm and nucleus were analyzed by Western blotting. β-actin was used as an internal control for the cytosolic fractions. Histone 3 was used as an internal control for the nuclear fractions. (B) The cells were stained with anti-p65 (green) antibody, nuclei were stained with DAPI (blue) to determine the nuclear localization of p65, the staining results were observed by confocal. Scale bar, 20mm. All results are presented as the means ± SD (n = 3) and are representative of three independent experiments. Differences between more than two groups were determined by two-way ANOVA with a Tukey’s post hoc test. ***p* < 0.01, ****p* < 0.001 and *****p* < 0.0001.


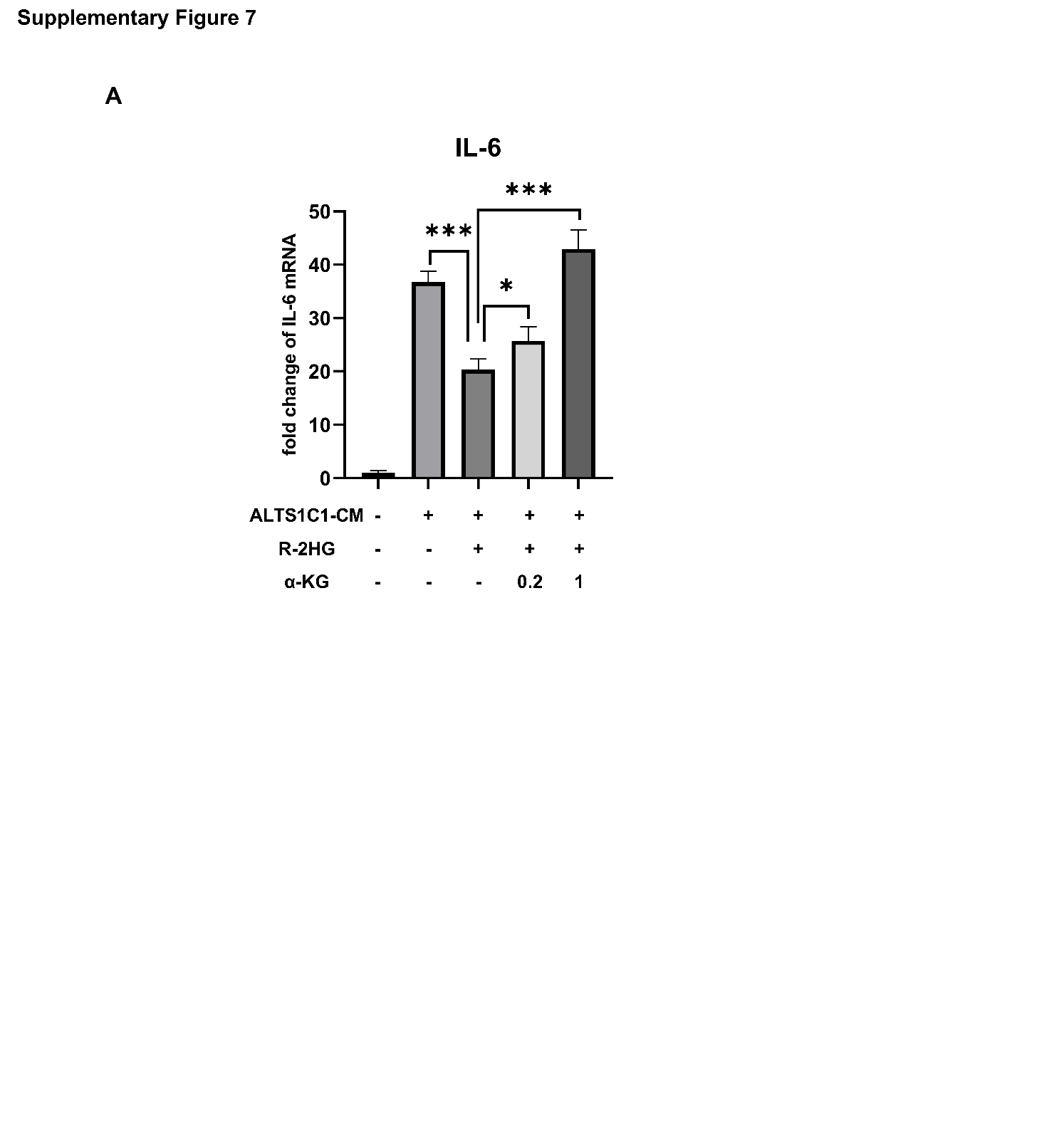


**Supplementary Figure 7.** **The addition of α-KG reverses the inhibitory effect of R-2HG on IL-6 in ALTS1C1-CM induced BV2 cells.** (A) In the conditioned medium stimulation system for ALTS1C1 glioma cells, R-2HG-treated BV2 cells were added with 0.2 mM and 1 mM α-KG, after incubation for 6h, the cells were assayed by qPCR for IL-6 mRNA expression levels. All results are presented as the means ± SD (n = 3) and are representative of three independent experiments. Differences between more than two groups were determined by one-way ANOVA with a Dunnett’s post hoc test. ***p* < 0.01, ****p* < 0.001 and *****p* < 0.0001 compared to CM+R-2HG.


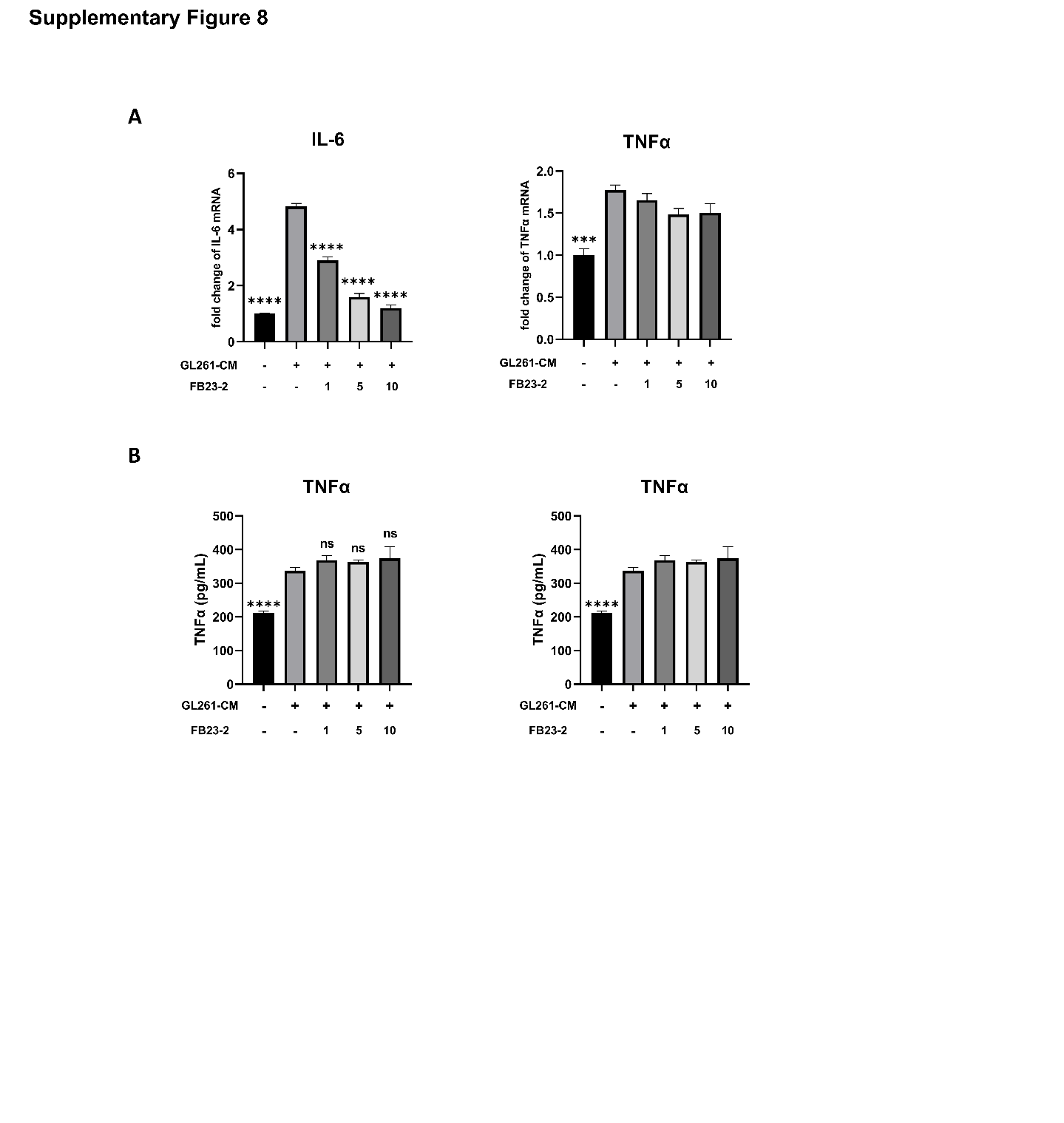


**Supplementary Figure 8.** **FTO inhibitor suppresses IL-6 expression in a dose-dependent manner.** Stimulated with conditioned medium from GL261 glioma cells, BV2 cells were added with 1mM, 5mM and 10mM FB23-2, respectively. (A) After incubation for 6 h, the mRNA levels of IL-6 and TNFα were analyzed by qPCR. (B) After incubation for 24 h, the protein levels of IL-6 and TNFα in supernatants were determined by ELISA. All results are presented as the means ± SD (n = 3) and are representative of three independent experiments. Differences between more than two groups were determined by one-way ANOVA with a Dunnett’s post hoc test. ***p* < 0.01, ****p* < 0.001 and *****p* < 0.0001 compared to CM.
